# Nucleotide lipid-based hydrogel as a new biomaterial ink for biofabrication

**DOI:** 10.1038/s41598-020-59632-w

**Published:** 2020-02-18

**Authors:** Bérangère Dessane, Rawen Smirani, Guillaume Bouguéon, Tina Kauss, Emeline Ribot, Raphaël Devillard, Philippe Barthélémy, Adrien Naveau, Sylvie Crauste-Manciet

**Affiliations:** 1 ARNA Laboratory, Inserm, U1212, CNRS 5320 (ChemBioPharm), University of Bordeaux, F-33000 Bordeaux, France; 2 Pharmaceutical Technology Department, University Hospital of Bordeaux, CHU de Bordeaux, Avenue de Magellan, 33604 Pessac, France; 3 Biotis, Inserm, U1026 University of Bordeaux 146 rue Léo-Saignat, Case 45 CEDEX 33076 F-, 33000 Bordeaux, France; 4 Department of Oral Medicine University Hospital of Bordeaux, CHU de Bordeaux, Place Amélie Rabat Léon, 33076 Bordeaux Cedex, France; 5 Center for Magnetic Resonance for Biological System UMR 5536, CNRS, University of Bordeaux 146 rue Léo Saignat, 33076 Bordeaux, France

**Keywords:** Biomaterials - cells, Supramolecular chemistry

## Abstract

One of the greatest challenges in the field of biofabrication remains the discovery of suitable bioinks that satisfy physicochemical and biological requirements. Despite recent advances in tissue engineering and biofabrication, progress has been limited to the development of technologies using polymer-based materials. Here, we show that a nucleotide lipid-based hydrogel resulting from the self-assembly of nucleotide lipids can be used as a bioink for soft tissue reconstruction using injection or extrusion-based systems. To the best of our knowledge, the use of a low molecular weight hydrogel as an alternative to polymeric bioinks is a novel concept in biofabrication and 3D bioprinting. Rheological studies revealed that nucleotide lipid-based hydrogels exhibit suitable mechanical properties for biofabrication and 3D bioprinting, including i) fast gelation kinetics in a cell culture medium and ii) shear moduli and thixotropy compatible with extruded oral cell survival (human gingival fibroblasts and stem cells from the apical papilla). This polymer-free soft material is a promising candidate for a new bioink design.

## Introduction

Biofabrication is a growing field in regenerative medicine and represents a promising tool for the construction of complex 3D structures^1–4^. Several approaches^5,6^ allow 3D-bioprinted construction to be achieved, with three main techniques: inkjet, laser and extrusion-based bioprinting^7^. Extrusion-based bioprinting (EBB) is one of the most widespread tools used in biofabrication, mainly due to its capacity to build large-volume constructs such as tissue or organ equivalents^5,8^. This technique offers a wide range of opportunities because it is suitable for various polymeric materials including scaffold-based (hydrogels, microcarriers, decellularised matrix components) as well as scaffold-free (cell aggregates) materials^5^.

Murphy *et al*.^1^ presented their concept of the “ideal material”, which needs to display several features, including printability, biocompatibility, good degradation kinetics, and favourable structural and mechanical properties, as well as material biomimicry. According to recent definitions^9^, a bioink can be defined as “*a formulation of cells that is suitable to be processed by an automated biofabrication technology*”. The presence of cells is a key element, and formulations that do not contain cells or involve the seeding of cells after printing do not qualify as bioinks. The term “biomaterial ink” (BmI) is then used. Consequently, this distinction will be used throughout our work.

Hydrogels are commonly used for the formulation of bioinks or BmIs and seem to be promising biomaterials because they are affordable, are easy to handle and can mimic the extracellular matrix^10^. Initial approaches of EBB used synthetic polymers such as polyethylene glycol (PEG) or gelatine methacrylate (GelMA) as scaffold materials, but they suffered from poor biological properties^11^. As an alternative, natural polymers derived from fibrous proteins or peptides have been developed due to their intrinsic biological qualities^12^. Among these polymers, alginate^13^, chitosan^14^, collagen^15^ and gelatine^16^ are the most studied. However, these polymers encounter several issues, such as poor mechanical properties^5^, the need for an external crosslinker to form a solid-like structure (e.g., reticulation of alginate with calcium cations^17^), insufficient rheological properties at physiological conditions preventing the printing process (e.g., pure gelatine presenting low viscosity at 37 °C^18^) and issues with long-term *in vivo* implantation (e.g., chitosan dissociating quickly at neutral pH^19^), as well as variability of sample properties from one batch to another (such as collagen^20^).

A major challenge in this field remains the development of bioinks and BmIs^21,22^ that satisfy both the physicochemical and biological requirements. Regarding mechanical properties, BmIs should form microstructures mimicking the cell’s native environment. In addition, bioink stiffness and porosity should be similar to the natural extracellular matrix to support cell growth and proliferation. These inks also have to exhibit shear-thinning behaviours to limit cellular stress resulting from the printing process^23^. More importantly, a bioink must neither be cytotoxic nor induce an immune or inflammatory response. In addition, if a bioink exhibits the capacity to be formulated in cell culture medium, it would be an advantage to ensure cell survival^24,25^.

Most of the synthetic or natural polymeric materials developed do not fully satisfy all the fundamental requirements for biofabrication at once, including both biological and physicochemical properties^26^. Despite the polymer-based materials reported so far, it remains challenging to develop a biocompatible material that can be printed under physiological conditions. A few studies have investigated non-polymeric supramolecular materials as potential BmIs or bioinks^27^. In this context, we hypothesised that low molecular weight gelators (LMWGs) could lead to non-toxic nucleotide lipid-based hydrogels suitable for biofabrication applications. As a supramolecular strategy involving biomolecules, this approach aims at developing biocompatible hydrogels under mild conditions^28^. Among LMWGs, bioinspired amphiphiles such as nucleotide lipids (NLs) and glycosyl-nucleoside lipids (GNLs) display interesting properties due to their chemical structure^28–31^. Composed of natural moieties such as nucleosides/nucleotides, sugars and aliphatic chains, these low molecular weight systems exhibit good biocompatibility, biostability and biodegradability. Their physical properties vary reversibly depending on conditions or stimuli (e.g., temperature^32^). Additionally, nucleotide lipid-based hydrogels formed by LMWGs^33,34^ represent a promising tool in biomedical applications since they display self-assembly abilities^35^ in complex aqueous media. The dynamic fibre networks in nucleotide lipid-based hydrogels provide soft scaffolds that mimic the extracellular matrix. Indeed, nucleotide lipid-based hydrogels are ideal physical gels because they possess the elastic behaviour of solids combined with the microviscous properties of fluids.

As a proof of concept, we demonstrated that a nucleotide lipid-based hydrogel could be used as a novel BmI or bioink for biofabrication. We have studied the physicochemical and biological properties of a bioconjugate^28,31^, including its rheological properties, printability in a culture medium, cell viability and *in vivo* tolerance.

## Materials and Methods

### Nucleotide lipid biomaterial ink formulation

The amphiphilic thymidine-based nucleotide lipid bioconjugate diC_16_dT reported in this study was synthesised by our team^28,30,31^, and hydrogel formulations were adapted from our previous studies.

Briefly, the synthesis of NLs containing a thymidine head group and 1,2-dipalmitoyl-*sn*-glycerol phosphate (diC_16_dT) is a direct synthesis achieved in a few steps starting from commercially available 5′-O-DMT-thymidine 3′-CE phosphoramidite. A standard synthetic procedure routinely used in oligonucleotide synthesis *via* phosphoramidite chemistry provides the thymidine-based NL in high yield^36^.

Regarding gel formation assays, diC_16_dT powder was dispersed in 0.9% sodium chloride aqueous solution (Fresenius Kabi, Bad Homburg vor der Höhe, Germany) or DMEM (Dulbecco’s modified Eagle’s medium) with low glucose, GlutaMAX™ and pyruvate Gibco® (Thermo Fisher Scientific, Waltham, Massachusetts, USA) at 3% or 4% (w/v) concentration.

As diC_16_dT is counter-cation dependent to form a gel^29^, it was prepared in 0.9% sodium chloride aqueous solution as a reference and in culture medium (DMEM) or DMEM enriched with sodium chloride. The ratio between culture medium and 0.9% sodium chloride aqueous solution was optimised to induce gel formation in the culture medium. By convention, the latter solution is named DMEM@+ in this manuscript. The osmolarity of DMEM@+ solution was controlled with a Type 15 osmometer (Fisher Scientific SAS, Illkirch Graffenstaden, France) to ensure its cell compatibility.

The samples were heated at 55 °C and stirred at 1000 rpm simultaneously for 30 minutes and then allowed to cool to room temperature. To determine the gel formation, all samples were turned upside-down; no flow under its own weight indicates stabilisation of the gel.

### Nucleotide lipid biomaterial ink characterisation

#### Determination of biomaterial ink mechanical properties by rheology

Rheological experiments were performed using a Malvern Kinexus Pro+ rheometer (Malvern Instrument SA, Worcestershire, United Kingdom) with a steel cone-plate geometry (diameter: 20 mm; angle: 1°). A solvent trap was used to prevent solvent evaporation and control the temperature. The lower plate was equipped with a Peltier temperature control system. Samples were studied at ambient (25 ± 0.01 °C) and physiological (37 ± 0.01 °C) temperatures.

Materials were placed in a low geometry under their gel state using a plastic spatula and allowed to rest 30 minutes before any experiment. All measurements were carried out within the linear viscoelastic regime (LVR), which was determined by amplitude strain sweeps from 0.01 to 100% deformation at an angular frequency of 1 Hz (6.283 rad/s). Elastic (also called storage modulus) (G’) and viscous (also called loss modulus) (G”) moduli were studied using a frequency sweep test ranging from 0.1 Hz to 10 Hz, and values were selected at 1 Hz.

To determine the transition temperature (Tsol-gel), a single-frequency stress-controlled temperature ramp was performed. This sol-gel transition temperature was determined at a 1 Hz frequency and a 1 Pa strain. The ramp ranged from 25 to 90 °C (heating rate of 2 °C/min).

Thixotropy was assessed by subjecting gels to low/high strain cycles. The gel was first subjected to a low strain (0.1%, into its LVR) for 10 minutes. Then, the sample was suddenly subjected to a higher strain (15%, beyond its LVR) for 2 minutes. Finally, the strain returned to the first step value (0.1%, into its LVR) until full recovery of the initial properties.

#### Biomaterial ink morphology

Transmission electron microscopy (TEM) experiments were performed with a Hitachi H7650 microscope (Hitachi Ltd., Chiyoda, Tokyo, Japan) linked to an ORIUS SC1000 11MPX (GATAN, Pleasanton, USA) camera run by Digital Micrograph software (GATAN)). Material ink samples (10 µL) were placed on carbon-coated copper grids at room temperature. The excess was removed, and the samples were dried for 10 minutes. Before TEM imaging, grids were negatively stained using Uranyl-Less solution (Delta Microscopies, Mauressac France).

#### Qualitative analysis of biomaterial printability

Biomaterial ink was first tested using a simple extrusion test with a syringe and thin needles (18 and 22 gauge). Briefly, BmI was prepared as described above and loaded in a syringe while liquid. After complete gelation, it was extruded with a syringe pump (Razel Scientific Instrument, Fairfax, USA) at a 4 µL/s flow rate, which is similar to the bioprinter flow rate.

#### Extrusion-based bioprinter

The Multiprint bioprinter used was a mechanical extrusion-based 3D printer designed by the Fablab Coh@bit (Technological University Institute of Bordeaux – IUT, Gradignan, France). Straight and non-bevelled Luer-lock needles of 18 and 22 gauge were employed. Computer-aided designs (CADs) were created with SketchUp software® (Trimble Inc., Sunnyvale, CA, USA). Multiprint was linked to BioPrinter® software, and one printing template was used: a lattice with 10 mm strands, 3 m inter-strand space and 2 × 2 mm^2^ pores (see Supplementary Fig. S1). Each layer of the lattice was composed of approximately 100 µL of hydrogel. Successful constructs had to possess at least 3 layers. The Bioprinter® software allowed adjustment of theoretical extrusion parameters (syringe volume, needle internal diameter and layer height) as well as real-time control to adapt ink extrusion during printing. The real-time parameter called the “extrusion coefficient” was expressed as a percentage.

#### Lattice characterisation

Five three-layer lattices were printed using Multiprint and the 3% diC_16_dT-DMEM@ + BmI in a six-well plate (one lattice per well). Whole lattices were then observed using a modular stereo microscope (MZ10F, Leica Microsystems, Germany) coupled with a Leica Application suite (V4.9.0, Leica Microsystems, Germany) at a magnification of x0.8. The distance between the vertical and horizontal strands of each lattice (see Supplementary Fig. S1) was determined using ImageJ software (1.52o, National Institute of Health, USA, Java 1.8.0_112). Lattices were also observed with a manual inverted microscope (DMI 3000B, Leica microsystems, Germany) coupled with the Leica Application Suite software (V3.8.0, Leica Microsystems, Germany) at a magnification of ×2.5. Surfaces of the upper and lower layers of each pore were then determined with the Leica software.

Lattice porosity (*Pt*, expressed as a percentage) was calculated using the formula F1, where *Vp* (mm^3^) represents the measured pore volume and *Vt* (mm^3^) represents the theoretical pore volume. Pores of a rather spherical shape were assimilated to truncated cones, and the F2 formula was used to determine their volumes (*Vp*) (*h* is the height of the lattice (mm), and *R*_1_ and *R*_2_ are the radius of the upper and lower layer, respectively (mm)) (see Supplementary Fig. S2). The theoretical pore volume *Vt* (mm^3^) with a parallelepiped form was calculated using the F3 formula (*h* is the height of the lattice (mm), *L* is the length (mm) and *l* is the width (mm) of the pore). The lattice was placed on a microscope slide, and the height was measured using a numerical calliper (Halmart RS PRO, 150 mm, 841–2518) with a precision of 10 µm. Finally, statistical analysis was performed on these data (described in the statistical section below).

$${\rm{F}}1:Pt=\frac{Vp}{Vt}\times 100$$

$${\rm{F}}2:Vp=h\times \frac{\pi }{3}\times ({{R}_{1}}^{2}+{{R}_{2}}^{2}+{R}_{1}\times {R}_{2})$$

$${\rm{F}}3:Vt=h\times L\times l$$

### Cell isolation and culture

Human gingival fibroblasts (HGFs) and stem cells from the apical papilla (SCAPs) were isolated from patients who underwent surgery at the University Hospital of Bordeaux (CHU de Bordeaux). All patients gave their informed oral consent according to the 1975 Declaration of Helsinki and denied having recently taken drugs that could affect connective tissue metabolism. Protocols were approved by the institutional committee for the protection of human subjects (Local Ethics Committee from academic hospital CHU de Bordeaux) and under the declaration for conservation and preparation of human body elements for scientific research number DC 2008–412 (French ministry of higher education, research and innovation).

#### Fibroblast isolation procedure

Gum surgical wastes were collected during surgery from healthy non-smoking patients with no history of periodontitis (n = 4). The epithelium was removed, and the remaining connective tissue was cut into small fragments, transferred and cultivated in cell culture flasks (37 °C, 5% CO_2_) in DMEM completed with 20% foetal calf serum (FCS), 100 UI/ml penicillin, 100 μg/ml streptomycin and 2.5 μg/ml amphotericin B (Gibco, Thermo Fisher Scientific, Waltham, MA, USA). The medium was changed every 3 days. After the first passage, HGFs were grown in DMEM and 10% FCS and used from passages 3 to 5.

#### SCAP isolation procedure

Freshly extracted third molars were stored in minimum essential medium alpha (alpha-MEM) supplemented with 20% FCS and penicillin (100 U/ml)/streptomycin (100 µg/ml) (Gibco, Thermo Fisher Scientific, Waltham, MA, USA). Briefly, dental apical papillae were digested in a mixture of 3 mg/ml collagenase and 4 mg/ml dispase (Sigma-Aldrich, Saint Louis, MO, USA) for one hour at 37 °C. The resulting cell suspension was filtered and cultivated in culture flasks (37 °C, 5% CO_2_). The medium was changed every two days until the cells reached confluence. After the first passage, SCAPs were grown in alpha-MEM, 10% FCS and used from passages 3 to 7.

### Cell-laden hydrogel lattice preparation

#### Bioink preparation

For the bioink preparation (i.e., cell-laden hydrogel), HGFs or SCAPs were mixed with 3% diC_16_dT-DMEM@+ BmI and placed in a 2 ml glass syringe; 3% diC_16_dT-DMEM@+ BmI was synthesised as previously reported. Briefly, either HGFs or SCAPs were trypsinised, counted, centrifuged and re-suspended in BmI to obtain a bioink concentration of 1.10^6^ cells/ml. The BmI was previously heated to 39 °C to make it more liquid to ease cell re-suspension and obtain homogenous bioinks.

#### Lattice 3D printing

Three- to five-layer lattices were printed in 6-well plates with the bioinks as previously reported (one lattice per well). The obtained lattices (n = 10 HGF bioink; n = 10 SCAP bioink) were used for *in vitro* experiments.

HGF lattices and SCAP lattices were incubated in DMEM, 10% FCS, and 1% PenStrep and in alpha-MEM, 10% FCS, and 1% PenStrep, respectively. The medium was changed every three days.

### *In vitro* experiments

#### Live-dead staining

A Live/Dead® assay (Thermo Fisher Scientific, Waltham, MA, USA) was performed on lattices at Day 1, 7 and 14 according to the manufacturer’s instructions. In living cells, the non-fluorescent substrate acetoxymethyl calcein (AMC) was cleaved into green fluorescent calcein by an intracellular esterase. Dead cells, whose membranes were compromised, allowed entry of the intercalating agent ethidium homodimer-1 (EDT-1), amplifying red fluorescence.

Samples were observed by confocal laser scanning microscopy (TCS SPE, CTR 6500, Leica, Wetzlar, Germany). Images were taken. The number of living and dead cells was counted from three images per sample. Cell viability was reported as a percentage with the following formula: the number of living cells/total number of cells.

#### Alamar blue assay

An Alamar Blue® assay (Thermo Fisher Scientific, Waltham, MA, USA) was performed to assess cellular activity at Day 1, 7 and 14. Alamar Blue® reagent is a cellular health indicator that uses the reducing power of living cells to quantitatively measure the spread of multilineage cells.

At each time point, three HGF and three SCAP lattices were incubated with Alamar Blue® reagent (resazurin) at 10% in culture medium for 2,5 hours at 37 °C. BmI lattices (without cells) were used as controls. Media were transferred to a new plaque, and absorbance was measured at 590 nm using a spectrometer (2030 Multilabel Reader VICTOR X3; PerkinElmer, Waltham, MA, USA) connected to WorkOut® software. The results were reported as the mean fluorescence ± standard deviation (SD) of three independent samples. After the test, bioink lattices and BmI lattices were washed twice, returned to medium and used for the following days. The medium was changed every three days.

### *In vivo* experiments

#### Animals

Protocols were based on the principles of Laboratory Animal Care of the National Society for Medical Research and approved by the Animal Care and Experiment Committee of the University of Bordeaux, France (Ref. 201701051243776-V2 APAFIS#8442). Experiments were performed following European recommendations for laboratory animal care (EU Directive 2010/63/EU for animal experiments). Four eight-week-old C57BL/6 female mice (Charles Rivers, France) were used for this study. Mice were anaesthetised with 4.5% isoflurane gas via an induction chamber and maintained throughout surgery with 2.5% isoflurane by using a facemask.

#### Subcutaneous injection

The surgical site was prepared by shaving, and the exposed skin was disinfected with betadine. On each of the four mice, two 500 µl BmI preparations were injected subcutaneously using a 1 ml syringe with an 18-gauge needle (Terumo Europe, Belgium). Each mouse received two bilateral injections.

#### Magnetic resonance imaging

Experiments were performed on a 7 T Bruker BioSpec system equipped with a gradient coil of 660 mT.m-1 maximum strength and 110 μs rise time. A volume resonator operating in quadrature mode was used for excitation (75.4 mm inner diameter, 70 mm active length), and a proton phased array (RAPID Biomedical GmbH) was used for signal reception (4 elements of 30 mm long around an elliptic cylinder housing: 19 × 25.5 mm).

A water-selective balanced steady-state free precession (WS-bSSFP) sequence was applied to image mouse abdomens and then measure BmI volumes over time. The following parameters were used: field-of-view (FOV): 25 × 22 × 20 mm, spatial resolution: 195 × 172 × 156 µm, TE/TR = 2/4 ms, reception bandwidth (BW): 75 kHz, and flip angle: 30 me. The following parameters were used: field-of-view (FOV): 25 × 2. Acquisitions were performed on 1.5% isoflurane-anaesthetised mice on the day of the injection and on Days 3, 8, 11, 14, 17, 21 and 24 post-injection.

### Statistical analysis

To handle reproducibility issues, experiments were performed in triplicate or more (n = 3). Rheological data were expressed as the mean ± SD. was used for all statistical analyses. Distribution normality was assessed with a Shapiro-Wilk test. Student’s t-test (2-tailed) with Fisher variance testing was performed to determine differences between groups.

For lattice strand distance, a Shapiro-Wilk test was used to assess distribution normality. If the distribution was normal, then Levene variance testing was performed. Depending on the Levene result, differences between groups were determined either by an ANOVA test (2-tailed) or a Kruskal-Wallis test (2-tailed). If the distribution was not normal, Kruskal-Wallis testing (2-tailed) was automatically performed.

Three tools were used to obtain results: XLstat® (Addinsoft, Paris, France) software, AnaStats website (http://www.anastats.fr/) and Astatsa website (https://astatsa.com/). Significance was set at *α* < 0.05.

For the Kruskal-Wallis test, H corresponded to the observed value, and cα was the critical value; the test was significant when H > cα. For ANOVA testing, a significant difference was set at *p* < 0.05, where *p* was the observed value.

## Results and Discussion

### Nucleotide lipid biomaterial ink formulation

**Figure 1:**
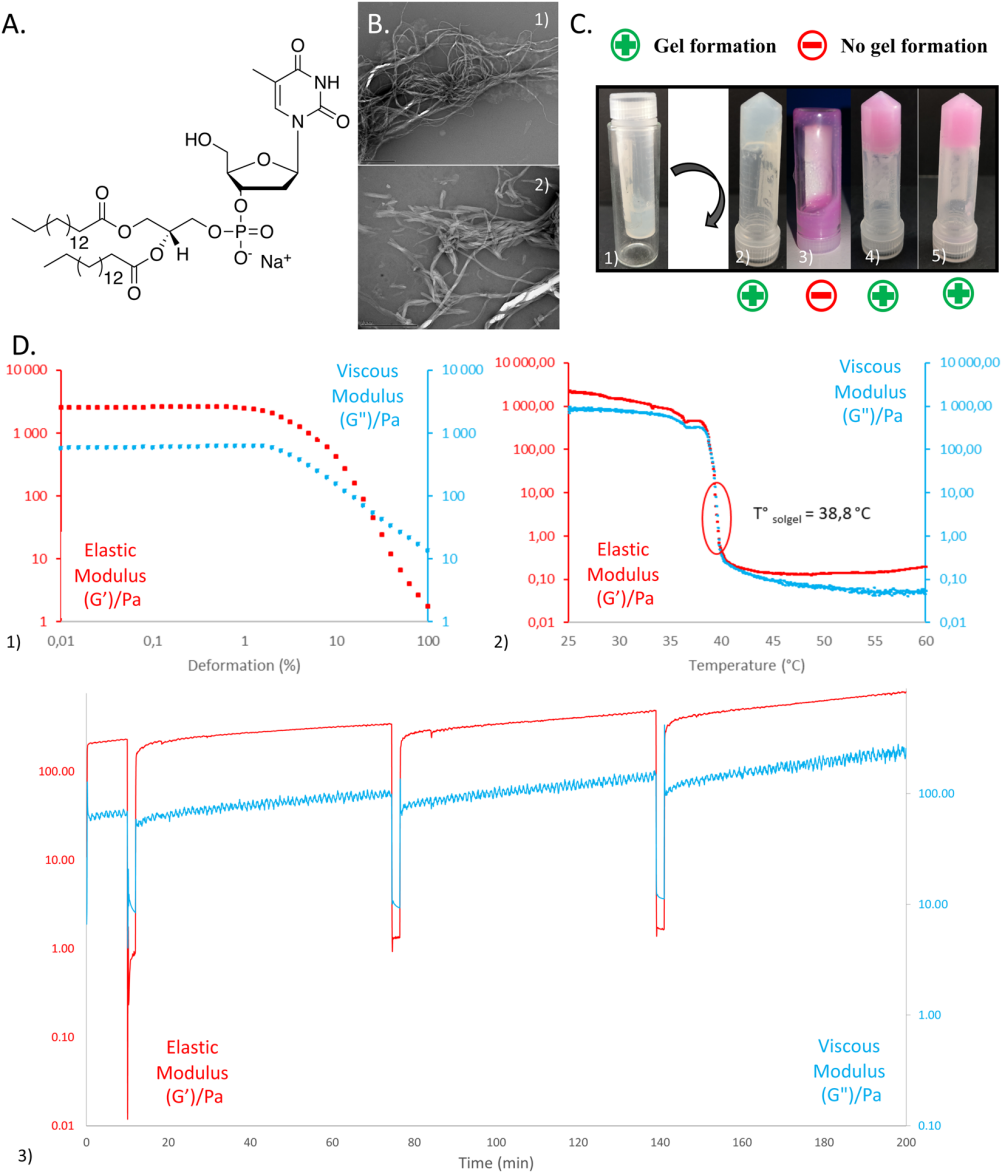
Biomolecular ink formulation and characterisation. (**A)** Chemical structure of low molecular weight hydrogelator thymidine head and 1,2-dipalmitoyl-sn-glycerol phosphate lipid chain diC_16_dT nucleotide lipid. (**B)** Inversion test to evaluate hydrogel gelation: **1)** and **2)** diC_16_dT at 3% (w/v) in 0.9% NaCl aqueous solution before and after reversal, respectively; **3)** diC_16_dT at 3% (w/v) in DMEM; **4)** diC_16_dT at 3% (w/v), in culture medium DMEM@+, **5)** diC_16_dT at 4% (w/v) in DMEM@+. (**C)** TEM images of diC_16_dT hydrogel (3% (w/v)) in culture medium (DMEM 123.5 mM NaCl final concentration): 1) scale of 2 µm length; 2) scale of 1 µm length. (**D)** Rheological results of diC_16_dT at 3% (w/v) in culture medium (DMEM@+): **1)** Linear viscoelastic regime at 25 °C, **2)** Transition temperature between 25 °C and 60 °C **3)** Step-strain experiment at 25 °C at a fixed angular frequency (6.283 rad/s). The gel was swept from 0.1% to 15% and then back to 0.1% strain. Key: DMEM@+ (DMEM containing NaCl 123.5 mM final concentration).

The selected bioconjugate molecule corresponds to an anionic thymidine-based NL bearing 1,2-dipalmitoyl-*sn*-glycerol phosphate as a lipid chain (Fig. 1A). Based on primary investigations, gel formation from this molecule in 0.9% sodium chloride aqueous solution was performed using the same protocol as previously described^28,30,31^. Identical formulation steps were applied in the case of culture medium DMEM.

Gel formation of diC_16_dT in 0.9% sodium chloride aqueous solution fully succeeded after heating (Fig. 1B 1) and 2)), but no gelation was observed in DMEM using the same protocol (Fig. 1B 3)). The sodium chloride content in DMEM (6.4 g/L) was insufficient to allow gelation. To successfully obtain a gel in the culture medium (Fig. 1B 4) and 5)), DMEM and 0.9% sodium chloride aqueous solution were mixed at an optimum ratio of 70/30% v/v (DMEM@+), giving a final NaCl concentration of 123.5 mM in the DMEM. The DMEM@+ osmolarity was measured at 311.5 ± 0.7 mOsm/l, allowing cell culture experiments and potential implantation *in vivo*. The 3% diC_16_dT-DMEM@+ BmI was observed by TEM imaging and showed a set of fibres with a thickness varying between 50 and 100 µm (Fig. 1C 1) and 2)) as previously observed in 0,9% NaCl^28^.

### Nucleotide lipid biomaterial ink characterisation

#### Rheological properties in culture medium

All measurements were carried out within the linear viscoelastic regime (LVR) (Fig. 1D 1)) at 0.1% deformation. In comparison to the 3% (w/v) diC_16_dT hydrogel prepared with 0.9% sodium chloride solution, the 3% (w/v) diC_16_dT hydrogel formed in culture medium (diC_16_dT-DMEM@+ BmI) weakened the BmI’s viscoelastic properties significantly (*p* = 0.002), as shown in Table 1, with a shear modulus G’ of 0.3 kPa in culture medium in comparison to ~2 kPa in sodium chloride solution. When the BmI was extruded, G’ and G” subsequently weakened for both conditions at 25 °C. Nevertheless, extrusion did not prevent the BmI from returning to its initial gel state and successfully completing the inversion test. When the temperature was increased to 37 °C, G’ and G” were increased in DMEM@+, with values of 675 ± 4.17 and 340 ± 2.30 Pa before extrusion, respectively. Similar to that at 25 °C, G’ and G” decreased after extrusion at 37 °C, with values of 153 ± 5 and 53 ± 6 Pa, respectively.

**Table 1:** Comparative shear moduli (G’ and G”) of diC_16_dT 3%- and 4%-based biomaterial ink in 0.9% NaCl or culture medium (DMEM@+) at 25 °C and 37 °C.

diC_16_dT 3%	Before extrusion		After extrusion	
Temperature	25 °C	37 °C	25 °C	37 °C
G’ (Pa)	362 ± 176 (1945 ± 549)*	675 ± 417	302 ± 230 (576 ± 171)*	153 ± 5
G” (Pa)	269 ± 144 (732 ± 200)*	340 ± 230	116 ± 29 (289 ± 105)*	53 ± 6
**diC**_**16**_**dT 4%**	**Before extrusion**		**After extrusion**	
Temperature	25 °C	37 °C	25 °C	37 °C
G’ (Pa)	2033 ± 181	3009 ± 773	1463 ± 567	2039 ± 398
G” (Pa)	742 ± 52	1356 ± 220	498 ± 177	640 ± 95

(*) diC16dT 3% based biomaterial ink G’ and G” in 0.9% NaCl.

The BmI also formed at higher diC_16_dT concentrations, i.e., 4% (Fig. 1B. 5)). As expected, the moduli increased simultaneously with the diC_16_dT concentration (see Table 1); at 4% diC_16_dT, G’ and G” increased to 2 kPa and 0,7 kPa, respectively, before extrusion and to 1,4 kPa and 0,5 kPa after extrusion at 25 °C. At physiological temperature, BmI’s viscoelastic properties were reinforced before and after extrusion, as shown by the moduli G’ and G” increasing beyond 2 kPa. Therefore, increasing the hydrogel strength could be useful to reinforce 3D-printed constructions. However, an increase in extracellular matrix stiffness can affect cell development and differentiation^37–39^. For example, stem cell differentiation either into neuron-like cells or into muscles is respectively modulated by stiffness values of the environment with low (0.1–1 kPa) and intermediate stiffness (8–17 kPa)^40^. For buccal mucosa HGF development, an equilibrium development was reached at a stiffness of 2 kPa^41^. Consequently, we decided to maintain the concentration at 3% diC_16_dT for the rest of our experiments.

Similar to that in the literature^28,30^, the 3% diC_16_dT-DMEM@+ BmI transition temperature (Tsol-gel) is approximately 40 °C (Fig. 1D 2)). This Tsol-gel in culture medium DMEM@+ allowed the addition of living cells for bioprinting purposes.

For biomedical use, thixotropy^42,43^ is a valuable tool allowing injectability and avoiding flap surgery for *in vivo* implantation (e.g., subcutaneous injection in mice^28,30^). This property makes crosslinker-free hydrogels interesting tools for bioprinting relative to natural or synthetic polymers, which often require an external polymerising agent. The diC_16_dT BmI exhibited thixotropic properties in 0.9% sodium chloride aqueous solution^28,30^. Our results showed that thixotropic properties were maintained in the culture medium DMEM@+ (Fig. 1D 3)) and at 37 °C. Moreover, the return to its initial gel state was almost immediate (1–2 minutes), confirming that 3% diC_16_dT-DMEM@+ BmI is a good potential candidate for biofabrication and future implantation.

#### 3D extrusion-based printing

Before using the Multiprint, BmI printability was evaluated by manual syringe pump extrusion. diC_16_dT was tested either in 0.9% sodium chloride aqueous solution or in DMEM@+. Both inks gained back a gel solid-like shape after extrusion.

**Figure 2:**
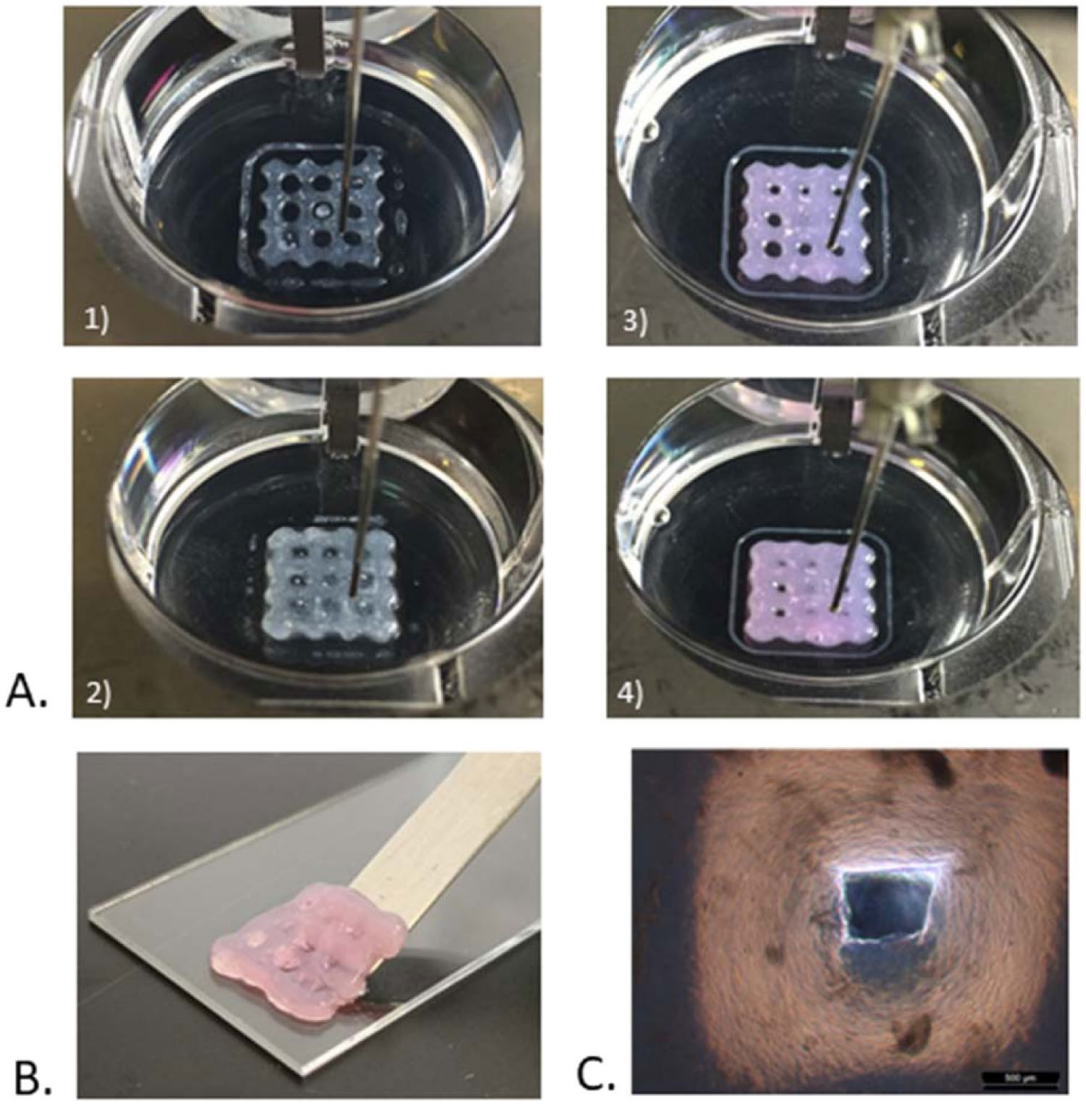
Biomolecular ink printability. (**A)** Bioprinted lattices of diC_16_dT hydrogel in 0.9% NaCl aqueous solution, **1)** 1 printed layer **2)** 5 printed layers; and in culture medium (DMEM@+) **3)** 1 printed layer, **4)** 5 printed layers. (**B)** Bioprinted lattice of diC_16_dT at 3% (w/v) in culture medium (DMEM@+) showing some flexibility. (**C**) Optical microscopy images of bioprinted lattice pores of diC_16_dT at 3% (w/v) in culture medium (DMEM@+). Magnification x2.5 (scale bars: 500 µm). Key: DMEM @+ (DMEM containing NaCl 123.5 mM final concentration).

After this first step, diC_16_dT-DMEM@+ BmI was printed using the Multiprint into a lattice matrix (Fig. 2A and movie). The lattice is a commonly used matrix in EBB, as it shows perfect compatibility with cell proliferation^5,44–46^. It was shown that cell growth is enhanced by pores, allowing a greater exchange surface between cells and culture medium. A pause of 2 minutes was taken between the extrusion of each layer to allow enough time for the hydrogel reconstruction and prevent lattice collapse. The extrusion coefficient was increased to counterbalance the higher viscosity of the diC_16_dT-DMEM@+ BmI compared to that of the diC_16_dT hydrogel formed in 0.9% sodium chloride aqueous solution.

The successful printability of diC_16_dT-DMEM@+ BmI was related to its rheological properties^18,47^, including shear thinning capacity and thixotropic properties, as previously highlighted. Printed lattices maintained their shape during the printing step and furthermore showed some flexibility (Fig. 2B).

Lattice characterisation was performed using microscopy imaging (Fig. 2C), which showed that the structures printed using diC_16_dT-DMEM@+ BmI were replicable. Indeed, the distances between each vertical and horizontal strand were statistically similar from one lattice to another (Kruskal-Wallis: H 3,83 < cα 9,49), with an average of 2,98 ± 0,14 mm and a theoretical distance of 3 mm (see data in Supplementary Table S1). The volume distribution of the 9 pores measured for each lattice and porosity was similar from one construction to another (ANOVA: p > 0.05; Kruskal-Wallis: H 4 < cα 9,49, respectively). The average pore volume was 1,63 ± 0,46 mm^3^ (theoretical volume 4,62 mm^3^), and the lattice total porosity (total pore volume/total lattice volume) was 8,61 ± 2,29% (theoretical porosity of 36%) (see all data in Supplementary Table S2). Except for one pore, the dimensions and porosity were maintained throughout all printed structures, showing the reproducibility of construction performed with the diC_16_dT-DMEM@+ BmI. These results appear favourable to the development and cellular growth for the manipulations incorporating cells within the BmI.

### Nucleotide lipid bioink formulation and *in vitro* cellular assays

The incorporation of cells into the 3% diC_16_dT-DMEM@+ BmI was easily performed by heating the hydrogel. Syringes were loaded with the obtained bioink, and the latter quickly returned to its gel state. Bioink incorporating either HGFs or SCAPs was easily extruded and successfully bioprinted into lattices.

#### In vitro cell assays

**Figure 3:**
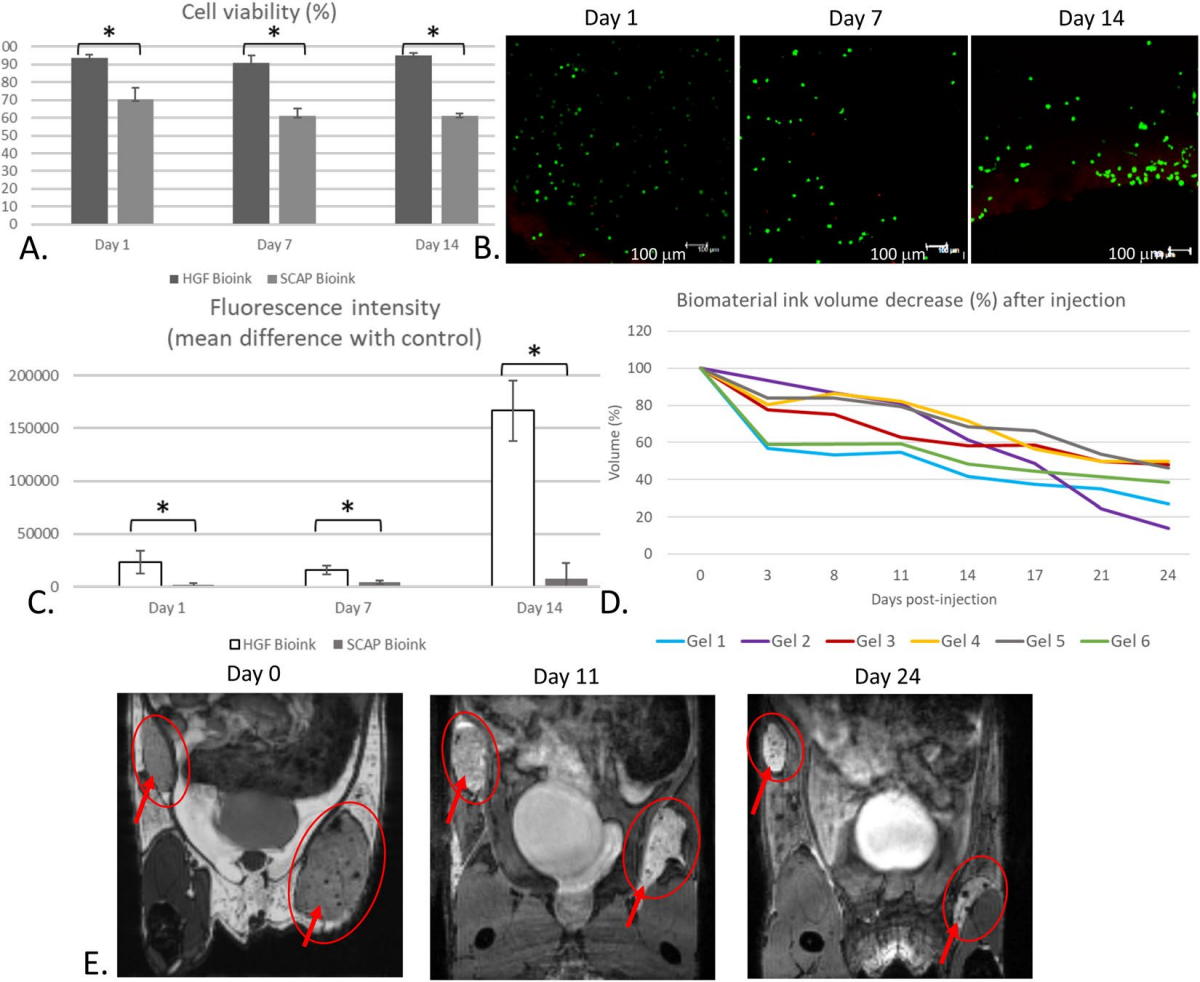
*In vitro* characterisation of bioprinted lattices and *in vivo* injection of diC_16_dT 3% DMEM@+ biomaterial ink. Evaluation of the cellular response of HGFs and SCAPs within lattices made by HGF and SCAP bioinks, respectively. (**A)** Viability percentage (*p < 0,05; n = 3). (**B)** Live/Dead staining of HGF proliferation in a lattice on days 1, 7 and 14. (**C)** Alamar Blue assay of lattices cultured for 1, 7 and 14 days (*p < 0,05, n = 3). Control was a lattice without cells. (**D)** Representation of injected diC_16_dT DMEM@+ biomaterial ink volume (reported in %) according to time. (**E**) Follow-up of two injections of diC_16_dT 3% biomaterial ink in a mouse at D0, D11 and D24. Key: DMEM@+ (DMEM containing NaCl 123.5 mM final concentration).

The viability of HGFs and SCAPs bioprinted in lattices is shown in Fig. 3A. Our results suggested that the printing process and hydrogel have low cytotoxic effects on HGFs but slightly more cytotoxicity on SCAPs. At Day 1, the SCAPs viability percentage was already lower than the HGF viability (70,5% versus 93,5%, respectively) (*p < 0,05; n = 3) (Fig. 3A). It is in accordance with previous studies showing that HGFs and SCAPs were resistant to shear stress and pressure during extrusion printing^48–50^. Moreover, SCAPs viability decreased while HGF viability remained high over time (Fig. 3A). These lower results concerning SCAPs could be explained by the fact that they are stem cells and are consequently more fragile than fibroblasts, complicating their ability to survive especially in the centre of lattices. Confocal microscopy observations with Live/Dead staining showed morphological changes (Fig. 3B). At Day 1, cells were round as a consequence of the recent cell encapsulation in the hydrogel (Fig. 3B). SCAPs and HGFs started to spread at Day 7, while some HGFs returned to their characteristic spindle shape (i.e., their normal shape in 2D culture) on Day 14 (Fig. 3B). The cell viability measures associated with cell observation enabled a clear understanding of this phenomenon, showing that the cells recovered as if they were cultured in flasks. All these results showed that cells, despite being totally encapsulated inside the gel and undergoing shear stress from bioprinting, were able to survive within the lattice for more than 21 days (see Supplementary Fig. S3). The Alamar Blue® assay, performed in triplicate for each time, showed a progressive increase in cellular activity (Fig. 3C). The HGF lattice fluorescence intensity when normalised to the control (i.e., lattice with the biomaterial ink only) displayed more than a three-fold increase at Day 14 when compared to Day 1 (Fig. 3C). The fluorescence of SCAP lattices was increased as well but at lower rates, which is in accordance with the decreased viability. Taken together, these results showed the cell compatibility of the diC_16_dT-DMEM@+ BmI, showing that this material provided enough porosity for oxygen and nutrients to reach cells despite the lattice thickness but not sufficient for long time culture. A bioreactor could be used to mimick the *in vivo* condition and increase follow-up time. Another solution could be to implement capillaries inside the construct prior to implantation. However, the results were slightly less obvious for stem cells. All results were statistically significant (p < 0.05). Progressive spreading and proliferation of cells within the gel could be due to modifications in viscoelastic properties after cell incorporation. These modifications were previously described for alginate, gelatine methacrylate and agarose hydrogels^51–53^. Indeed, long-term variations of construct properties were mainly due to cell proliferation and migration, leading to hydrogel matrix remodelling. Overall, the printing results are promising and will be improved by other studies, with varying ratios, growth factors, structural proteins or nutrients.

#### *In vivo* nucleotide lipid biomaterial ink biodegradability

Previous histological studies have been performed in our lab and showed that diC_16_dT hydrogels formed in 0.9% sodium chloride aqueous solution demonstrated minimal inflammation and fibrosis^28^. To study the impact of biopriting process on gel structure and the *in vivo* behaviour, we decided to perform a MRI follow up. Out of the eight diC_16_dT-DMEM@+ BmI injections in mice, two could not be followed (non-subcutaneous injection) at D0. The WS-bSSFP MRI sequence^54^ was used to follow up the remaining six samples. After subcutaneous injection, a 20 to 40% decrease in volume was observed in D3, followed by a gradual decrease until 50–85% of the initial volume at D24 (Fig. 3D,E). Statistical analysis performed on BmI percentage degradation reported a comparable degradation from one gel to another (Kruskal-Wallis: H 5 < cα 11,0705) (see Supplementary Tables S3 and S4). No diffusion in other tissues was observed. Moreover, no evidence of inflammation was detected in all mice during monitoring, showing *in vivo* tolerance. It was voluntarily decided to study injectable implantation instead of flap surgery to limit inflammation linked to the procedure and compared this experiment to previous results. As a first step for *in vivo* evaluation, this experiment supported the evidence that a diC_16_dT-DMEM@+ BmI is biodegradable and revealed the diC_16_dT-DMEM@+ formulation to be compatible for future graft implantation. Further studies will be performed to determine the *in vivo* behaviour of bioprinted cellularized lattices.

## Conclusion

We have reported a novel nucleotide lipid-based material featuring suitable mechanical and biological properties for different biofabrication applications, including injection and 3D bioprinting. To our knowledge, this is the first example of materials using a nucleotide lipid-based LMWG hydrogel allowing (i) bioprinting under physiological conditions with culture medium and cells as well as (ii) good *in vivo* tolerance when injected subcutaneously. Physicochemical investigations performed on diC_16_dT gels solubilised in DMEM enriched with NaCl (DMEM@+) revealed that this molecule was able to stabilise a gel compatible with the survival and proliferation of human gingival fibroblasts and stem cells from apical papilla. Thus, the rheological properties of the 3% hydrogel showed interesting features for EBB, such as a Tsol-gel superior to 37 °C, allowing cell culture and thixotropy to provide a biomaterial free of external crosslinkers. Furthermore, diC_16_dT-DMEM@+ displayed *in vivo* compatibility and gradual biodegradation over time. In conclusion, the results highlighted the workability, printability and biocompatibility of nucleotide lipid-based hydrogels, as bioprinted cells exhibit viability and proliferation. This preliminary study was designed as a starting point for a larger project concerning tissue engineering-based regeneration of connective tissue. Further studies will be performed in order to specify the *in vivo* behaviour of bioprinted and cellularised lattices.

*In fine*, the use of lipid self-assemblies stabilised by a nucleotide moiety could represent a powerful strategy for bioink engineering, opening a new route to produce crosslinker-free scaffolds suitable for different biofabrication applications. In the long term, this concept could be applied to any connective tissue reconstruction by changing the type of cells.

## Supplementary information

Supplementary information.

Video: Bioprinted lattice using a nucleolipid based gel.
