# Supplementary material for: Nucleotide lipid-based hydrogel as a new biomaterial ink for biofabrication
Source: Sci Rep. 2020 Feb 18;10:2850. doi: 10.1038/s41598-020-59632-w (PMC7029012; doi:10.1038/s41598-020-59632-w)
Supplement: Supplementary file 1 — Supplementary information. [file 41598_2020_59632_MOESM1_ESM.docx]

**Supplementary information**

**Nucleotide lipid-based hydrogel as a new biomaterial ink for biofabrication**

*Bérangère Dessane*†*, Rawen Smirani*†*, Guillaume Bouguéon*†*, Tina Kauss, Emeline Ribot, Raphaël Devillard, Philippe Barthélémy*, Adrien Naveau, Sylvie Crauste-Manciet*

(†) B. Dessane, R. Smirani and G. Bouguéon have contributed equally to this work as first authors.

(*) Corresponding author

**Lattice characterisation**


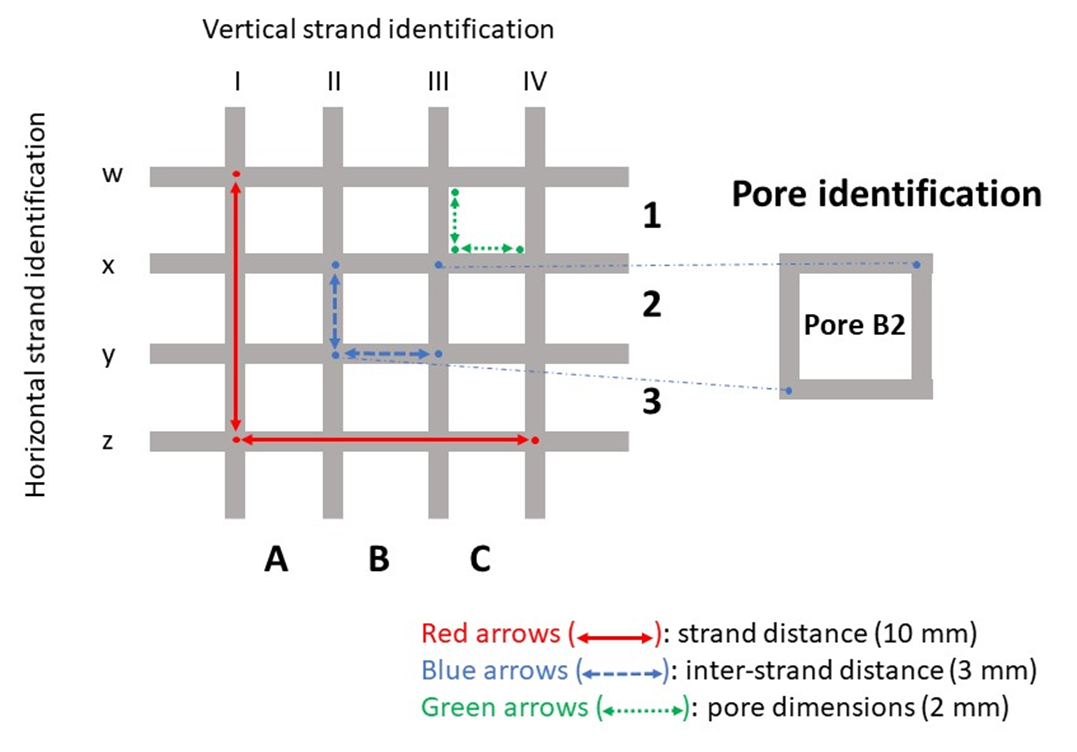


**Supplementary Figure S1. Determination of the strand distance (red arrows), inter-strand distance (blue arrows) and pore dimension width and letter of lattices.** Schematic representation of the printed lattices and explanation on strand and pore identification. Vertical and horizontal strands are denoted by a Roman numeral (i.e., I; II; III; IV) and a lowercase letter (i.e., w; x; y; z), respectively. Pores are denoted by the association of a capital letter and an Arabic numeral (e.g., pore A.1; B.2; C.3…). Theoretical strand distances and pore dimensions are specified in brackets.


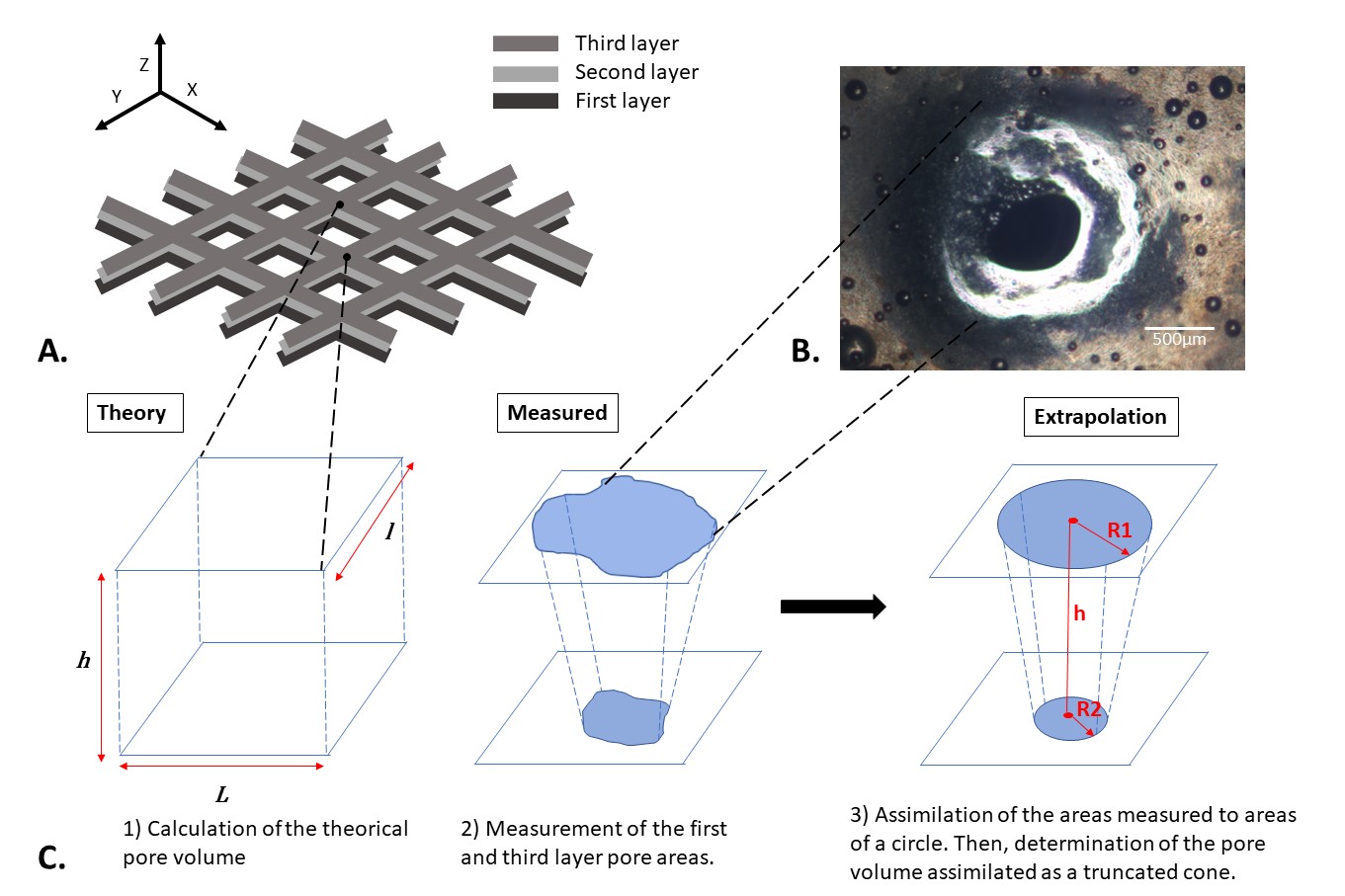


**Supplementary Figure S2. Pore volume determination. A.** Schematic representation of a three-layer printed lattice; **B.** Microscopic image of one pore of a three-layer lattice (magnification x2.5); **C.** Process used to determine the pore volume. 1) The theoretical pore volume is calculated using the computer-aided design parameters. 2) The area of the first and third layers of printed lattices is determined using Leica application software. 3) Then, these areas are assimilated to circle areas. The pore volume is calculated using the F2 formula. The pore theoretical volume is calculated using the F3 formula (see the Lattice characterization section in the Material and Methods for formulas).

**Supplementary Table S1.** Average distances between each vertical and horizontal strand for 5 lattices and statistical analysis.

μ = average; σ = standard deviation;

|  |  | **Lattice 1** | | **Lattice 2** | | **Lattice 3** | | **Lattice 4** | | **Lattice 5** | |
| --- | --- | --- | --- | --- | --- | --- | --- | --- | --- | --- | --- |
| **Inter-strand measured** | | **μ (mm)** | **σ** | **μ (mm)** | **σ** | **μ (mm)** | **σ** | **μ (mm)** | **σ** | **μ (mm)** | **σ** |
| **Vertical strand** | **I-II** | 2965 | 18 | 3036 | 20 | 3012 | 8 | 3034 | 24 | 3212 | 11 |
|  |  |  |  |  |  |  |  |  |  |  |  |
|  |  |  |  |  |  |  |  |  |  |  |  |
|  |  |  |  |  |  |  |  |  |  |  |  |
|  | **II-III** | 2986 | 26 | 2763 | 0 | 2750 | 24 | 3038 | 13 | 2759 | 13 |
|  |  |  |  |  |  |  |  |  |  |  |  |
|  |  |  |  |  |  |  |  |  |  |  |  |
|  |  |  |  |  |  |  |  |  |  |  |  |
|  | **III-IV** | 2941 | 19 | 3086 | 24 | 3096 | 25 | 2893 | 20 | 3200 | 18 |
|  |  |  |  |  |  |  |  |  |  |  |  |
|  |  |  |  |  |  |  |  |  |  |  |  |
|  |  |  |  |  |  |  |  |  |  |  |  |
| **Horizontal strand** | **W-X** | 2852 | 9 | 2797 | 11 | 2833 | 7 | 2843 | 25 | 2898 | 32 |
|  |  |  |  |  |  |  |  |  |  |  |  |
|  |  |  |  |  |  |  |  |  |  |  |  |
|  |  |  |  |  |  |  |  |  |  |  |  |
|  | **X-Y** | 2969 | 9 | 3067 | 11 | 3022 | 17 | 2928 | 21 | 3052 | 69 |
|  |  |  |  |  |  |  |  |  |  |  |  |
|  |  |  |  |  |  |  |  |  |  |  |  |
|  |  |  |  |  |  |  |  |  |  |  |  |
|  | **Y-Z** | 3315 | 19 | 3133 | 15 | 3201 | 11 | 2937 | 60 | 3015 | 41 |
|  |  |  |  |  |  |  |  |  |  |  |  |
|  |  |  |  |  |  |  |  |  |  |  |  |
|  |  |  |  |  |  |  |  |  |  |  |  |

**Supplementary Table S2.** Calculation of pore volume porosity and statistical analysis for 5 printed and theoretical lattices.

|  |  | **Lattice 1** | **Lattice 2** | **Lattice 3** | **Lattice 4** | **Lattice 5** | **Theoretical lattice** |
| --- | --- | --- | --- | --- | --- | --- | --- |
| ***Pore Volumes (mm3)*** | | | | | | | |
| **Pores** | **A1** | 1.45 | 1.14 | 0.77 | 1.94 | 1.83 | *4.62* |
|  | **A2** | 1.21 | 2.23 | 1.25 | 3.37 | 2.88 | *4.62* |
|  | **A3** | 4.51 | 1.38 | 1.57 | 3.36 | 2.71 | *4.62* |
|  | **B1** | 1.22 | 0.84 | 0.44 | 0.00 | 2.29 | *4.62* |
|  | **B2** | 1.33 | 0.26 | 1.01 | 2.40 | 2.47 | *4.62* |
|  | **B3** | 1.10 | 0.88 | 0.51 | 1.73 | 2.26 | *4.62* |
|  | **C1** | 1.57 | 0.24 | 0.34 | 1.73 | 1.83 | *4.62* |
|  | **C2** | 2.41 | 1.37 | 1.76 | 1.42 | 2.00 | *4.62* |
|  | **C3** | 0.77 | 2.16 | 2.86 | 1.15 | 1.50 | *4.62* |
|  | **Total pore volume** | 15.58 | 10.51 | 10.51 | 17.09 | 19.79 | *41.58* |
|  | **Average pore volume** | 1.73 | 1.17 | 1.17 | 1.90 | 2.20 | *4.62* |
|  | **Standard deviation** | 1.14 | 0.72 | 0.81 | 1.06 | 0.45 | *0.00* |
| **Average lattice pore volume** | | 1.63 | | | | |  |
| **Standard deviation** | | 0.46 | | | | |  |
| ***Lattice Height (mm)*** | | | | | | | |
| **Height (mm)** | | 1.53 | 1.57 | 1.55 | 1.48 | 1.50 | *1.155* |
| ***Strand Lengths (mm)*** | | | | | | | |
| **Strands** | **I** | 10.35 | 10.19 | 10.02 | 10.60 | 10.34 | *10* |
|  | **IV** | 10.63 | 10.32 | 10.40 | 10.62 | 10.53 | *10* |
|  | **w** | 10.94 | 10.59 | 10.91 | 11.07 | 10.86 | *10* |
|  | **z** | 10.65 | 10.89 | 10.86 | 11.17 | 10.83 | *10* |
| ***Lattice volume without pores (mm3)*** | | | | | | | |
| **Volume (mm3)** | | 170.17 | 165.53 | 166.88 | 177.31 | 170.09 | *115.5* |
| ***Porosity %*** | | | | | | | |
| **Porosity %** | | 9.16 | 6.35 | 6.30 | 9.64 | 11.63 | *36* |
| **Average porosity %** | | 8.61 | | | | |  |
| **Standard deviation** | | 2.29 | | | | |  |
|  |  |  |  |  |  |  |  |

**In vivo biodegradability**

**Supplementary Table S3.** Monitoring of diC_16_dT DMEM@ + biomaterial ink (BmI) volumes (expressed in mm^3^) injected in mice according to time.

un. = unmeasurable

|  | **Gel volume (mm^3^)** | | | | | | | |
| --- | --- | --- | --- | --- | --- | --- | --- | --- |
| **Time (days)** | **Gel 1** | **Gel 2** | **Gel 3** | **Gel 4** | **Gel 5** | **Gel 6** | **Average** | **Standard deviation** |
| **0** | 226.70 | 149.15 | 154.13 | 124.48 | 121.41 | 206.21 | 163.68 | 43.37 |
| **3** | 128.89 | un. | 119.55 | 99.89 | 101.68 | 121.21 | 114.25 | 12.79 |
| **8** | 120.68 | 129.54 | 115.54 | 107.66 | 101.68 | un. | 115.02 | 10.90 |
| **11** | 124.04 | 120.99 | 97.01 | 102.11 | 96.43 | 122.37 | 110.49 | 13.30 |
| **14** | 94.58 | 91.41 | 89.54 | 88.92 | 82.93 | 100.06 | 91.24 | 5.77 |
| **17** | 84.53 | 72.72 | 90.12 | 70.21 | 80.45 | 91.89 | 81.65 | 8.91 |
| **21** | 79.51 | 36.33 | 76.47 | 61.81 | 65.16 | un. | 63.85 | 17.09 |
| **24** | 60.90 | 20.87 | 73.74 | 62.00 | 55.97 | 79.58 | 58.84 | 20.57 |

**Supplementary Table S4.** Monitoring of diC_16_dT DMEM@ + biomaterial ink (BmI) degradation (expressed in %) injected in mice according to time.

un. = unmeasurable

|  | **Percentage of BmI degradation (%)** | | | | | | | |
| --- | --- | --- | --- | --- | --- | --- | --- | --- |
| **Time (days)** | **Gel 1** | **Gel 2** | **Gel 3** | **Gel 4** | **Gel 5** | **Gel 6** | **Average** | **Standard deviation** |
| **0** | 0.00 | 0.00 | 0.00 | 0.00 | 0.00 | 0.00 | 0.00 | 0.00 |
| **3** | 43.15 | un. | 22.44 | 19.75 | 16.25 | 41.22 | 28.56 | 12.65 |
| **8** | 46.77 | 13.15 | 25.04 | 13.51 | 16.25 | un. | 22.94 | 14.16 |
| **11** | 45.28 | 18.88 | 37.06 | 17.97 | 20.58 | 40.66 | 30.07 | 12.28 |
| **14** | 58.28 | 38.71 | 41.91 | 28.57 | 31.69 | 51.47 | 41.77 | 11.42 |
| **17** | 62.71 | 51.24 | 41.53 | 43.59 | 33.74 | 55.44 | 48.04 | 10.46 |
| **21** | 64.93 | 75.64 | 50.39 | 50.34 | 46.33 | un. | 57.53 | 12.36 |
| **24** | 73.14 | 86.01 | 52.16 | 50.19 | 53.90 | 61.41 | 62.80 | 14.13 |

**Supplementary Figure S3.** In vitro characterisation of bioprinted lattices of diC16dT 3% DMEM@+ biomaterial ink. Cellular response of HGFs within lattices made by HGF bioinks at different sample locations. Live/Dead staining of HGF proliferation in the lattice on days 1, 7, 14 and 21. Living cells appear in green, the red colour observed is due to the biomaterial ink autofluorescence.


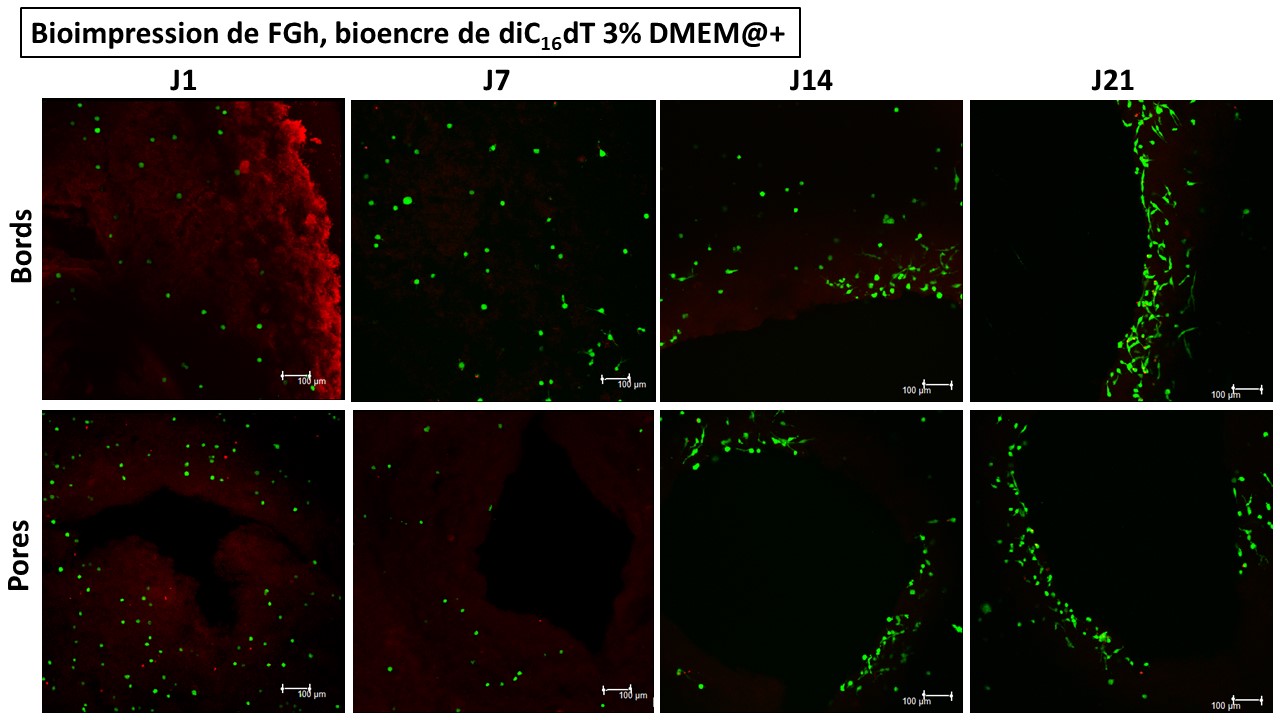


Edge

Pore

D1

D7

D14

D21
